# Examining the challenges posed to parents by the contemporary screen environments of children: a qualitative investigation

**DOI:** 10.1186/s12887-018-1106-y

**Published:** 2018-04-07

**Authors:** Emma Solomon-Moore, Joe Matthews, Thomas Reid, Zoi Toumpakari, Simon J. Sebire, Janice L. Thompson, Deborah A. Lawlor, Russell Jago

**Affiliations:** 1 Department for Health, University of Bath, Claverton Down, Bath, BA2 7AY UK; 2 Centre for Exercise, Nutrition & Health Sciences, School for Policy Studies, University of Bristol, 8 Priory Road, Bristol, BS8 1TZ UK; 3 School of Sport, Exercise and Rehabilitation Sciences, University of Birmingham, B15 2TT, Birmingham, UK; 4 MRC Integrative Epidemiology Unit at the University of Bristol, Oakfield House, Oakfield Grove, Bristol, BS8 2BN UK; 5 Population Health Science, Bristol Medical School, University of Bristol, Canynge Hall, Whiteladies Road, Bristol, BS8 2PS UK

**Keywords:** Parents, children, screen-viewing, qualitative, interview

## Abstract

**Background:**

The ubiquity of technology in modern society has led to the American Academy of Pediatrics adapting their screen-viewing (SV) recommendations for children. The revised guidelines encourage families to identify an appropriate balance between SV and other activities. The aims of this study were to explore parents’ views of their child’s SV time and how important it is for families to achieve a ‘digital balance’.

**Methods:**

Semi-structured telephone interviews were conducted with 51 parents of 8–9-year-old children, between July and October 2016. Inductive and deductive content analyses were used to explore parents’ perceptions of their child’s level of SV (low, medium, high), how parents feel about child SV, and the importance placed on achieving a digital balance. Parent report of child SV behaviours on weekdays and weekend days were assessed via questionnaire.

**Results:**

Interview data revealed that because SV is considered the ‘norm’, parents struggle to limit it, partly because they want their children to be equipped for the modern technological world. While most parents believe SV to have negative effects on children, parents also report advantages to SV. Many parents feel that not all SV is equal, with tablets considered worse than television because of the isolated nature of activities, and educational SV considered more beneficial than non-educational SV. Most parents feel it is important for their family to achieve a digital balance, primarily to spend more quality family time together. Large variation was observed in parents’ descriptions of child SV time on weekdays and weekend days.

**Conclusions:**

Parents recognise the importance of digital balance but want their children to fit into the ever-advancing digital world. Parents do not treat all SV equally. Watching television and engaging in educational SV may be encouraged, while ‘playing’ on tablets is discouraged. These findings highlight the challenge faced by researchers and policy makers to help families achieve a digital balance, and strategies are needed to support parents to plan child SV time.

**Electronic supplementary material:**

The online version of this article (10.1186/s12887-018-1106-y) contains supplementary material, which is available to authorized users.

## Background

Screen-viewing (SV) is associated with increased metabolic risk [1], decreases in psychological wellbeing [2], and reduced academic performance among children [3]. As SV behaviours track from childhood to adulthood [4], it is important for researchers and policy makers to find ways to help families manage children’s SV. The majority of SV research to date has focused on television viewing [5–10]. However, children are now engaging with a wide variety of SV devices (e.g., tablets, smartphones, games consoles, laptops), often using two or more devices simultaneously (multi-SV) [11], using online platforms (e.g., YouTube, Netflix), and engaging in SV for a greater number of purposes (e.g., homework, social interaction, entertainment, relaxation) [12]. Due to the ubiquity of technology in modern society, previous SV recommendations by the American Academy of Pediatrics (AAP) [13], suggesting children’s SV should not exceed two hours per day, have been revised. A ‘Family Media Use Plan’ is now encouraged, helping families to identify an appropriate balance between SV and other activities with no set SV thresholds. Instead, plans are individualised to a child’s age, health, temperament and development stage [12]. Therefore, it is important for researchers to understand the family dynamics associated with child SV, to help inform the communication of such recommendations and comprehend the environment in which families are likely to implement any SV intervention.

Parents are an important influence on their child’s SV, acting as the ‘gatekeepers’ to SV time [14]. Parents influence child SV behaviours by providing children with screen devices [15], setting SV rules [16, 17], as well as through their attitudes toward SV [17], and their own SV behaviour [18]. For example, findings from the baseline B-Proact1v study suggest that parents’ SV time is positively associated with their child’s SV time [18]. These data also revealed that despite reporting high levels of child SV [18], parents are generally not concerned about their child’s SV [19]. A study of parents of pre-school-aged children from the US found that while most parents were aware of the previous AAP SV guidelines, not all of them restricted child SV to less than two hours [20]. These findings suggest that simply providing parents with information on SV recommendations may not be sufficient to change behaviours, and it is possible that parents are uncertain about the importance of why guidelines exist. Further research is, therefore, warranted to explore parents’ knowledge and perceptions of the impact of SV on their child’s health and development, to understand if communication of the negative effects of SV needs to be clearer, or whether communication methods alone are sufficient to elicit behaviour change.

Previous studies have suggested that parents experience feelings of internal conflict regarding their child’s SV behaviour [12]. A systematic review of 21 studies, which examined parental perceptions regarding healthy behaviours for young children (from birth to 12 years of age, 62% aged < 5 years), revealed that while some parents often feel guilty about their child’s SV, perceiving SV as a barrier to physical activity, they also see it as an educational tool, and as such have little desire to restrict SV [21]. Two Canadian studies conducted focus groups with parents (85% mothers) of children aged up to four years in 2013 [22] and parents (66% mothers) of 5–17 year-olds in 2015–2016 [23], finding that despite being aware of the negatives of SV, some parents value SV as a behaviour management strategy, using the provision or restriction of SV time to reward or punish child behaviour, or by using SV as an ‘electronic babysitter’ so parents can complete household tasks [22, 23]. To date, there has been a lack of research focused on understanding parents’ internal conflict regarding child SV for children of primary-school age, and studies from the UK. Thus, in order to develop effective ways to help families recognise the importance of achieving a ‘digital balance’, defined as a lifestyle that includes SV but also includes a good balance of other activities and time away from SV, there is a need for studies to examine the internal conflict experienced by parents regarding their feelings toward child SV behaviour.

The aim of this qualitative study was to examine mothers’ and fathers’ views on their 8–9-year-old child’s SV behaviour, in terms of the internal conflict felt by parents regarding the positive and negative aspects of SV, and the importance placed on achieving a ‘digital balance’. A secondary aim was to explore how parents describe their child’s SV, to understand what level of SV time parents consider to be low, medium, or high.

## Methods

This study utilised data from the B-Proact1v study, which aimed to examine factors associated with children’s and parents’ physical activity and SV behaviours. The study has been described in detail elsewhere [18, 24, 25]. Briefly, in 2012 and 2013, data were collected from 1299 Year 1 children (5–6 years old) and at least one of their parents, from 57 primary schools across Bristol and the surrounding area, UK. Between March 2015 and July 2016, all 57 schools were approached to re-join the study when the children were in Year 4 of primary school (8–9 years). A total of 47 schools agreed to take part, and data were collected from 1223 families. Semi-structured telephone interviews were conducted between July and October 2016 with a sub-sample of 51 parents who participated in the study when their child was in Year 4 of school (age 8–9). In the main study, children and parents were asked to complete a questionnaire and wear an accelerometer to objectively measure physical activity and sedentary behaviour. Child height, weight and blood pressure were also objectively measured. Child height and weight were used to derive an age-adjusted body mass index (BMI) z-score using the 1990 UK child growth reference [26]. Families with complete data for all measures (child and parent accelerometer and questionnaire data, child height, weight and blood pressure) were eligible for inclusion in the interview sample (*N* = 625).

### Purposive sampling of the interview sample

**Fig. 1:**
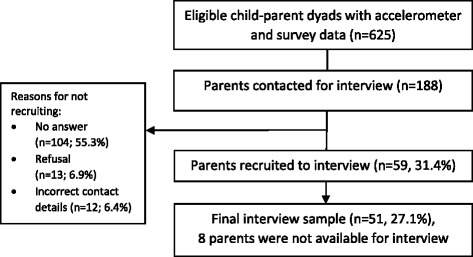
Study flow of participants

For the purposes of interview variability, the sample was stratified according to the child’s gender, accelerometer-assessed moderate-to-vigorous-intensity physical activity (MVPA) minutes per day, and daily time spent sedentary. This stratification produced eight sub-groups (one = low MVPA, low SED boys; and eight = high MVPA, high SED girls), which guided a purposive sample. The order in which parents were invited to take part was randomised within each sub-group. In total, 188 contact attempts were made by telephone, from which 59 parents (31.4%) initially agreed to participate in an interview, and 51 (27.1%) completed an interview (Fig. 1). Interviews were conducted at the interviewee’s convenience ((37 during weekday daytimes (72.5%), 13 during weekday evenings (25.5%), and 1 on a weekend evening (2%)) and were audio-recorded. Participants received a £10 voucher as a thank you for their participation in the study. Interviews were conducted until theoretical saturation was reached for the entire sample and within the eight sub-groups. The study received ethical approval from the School for Policy Studies Ethics Committee at the University of Bristol, and written parent consent was received for all participants [27].

### Interviews

Development of the interview guide (provided in Additional file 1) was informed by gaps in existing knowledge and guided by the Year 1 B-Proact1v quantitative and qualitative findings [18, 19, 25, 28–37]. Questions explored parents’ views of their 8–9-year-old child’s SV and physical activity behaviours, strategies for managing these behaviours, understanding what has changed regarding these behaviours, and the importance placed on families achieving a digital balance. Parents were also asked to describe their child’s level of SV on weekdays and weekend days as low, medium, or high. The interview guide employed open-ended questions posed in a non-leading manner to allow participants to shape the direction of the interview, and issues that emerged were probed. Interviews were conducted by two female researchers (qualified to at least MSc level), who were trained in conducting qualitative interviews. The two researchers met frequently throughout data collection to discuss and review the data to ascertain when theoretical saturation was reached.

### Accelerometer data collection

Participants wore a waist-worn ActiGraph wGT3X-BT accelerometer for five days, including two weekend days. Accelerometer data were processed using Kinesoft (v3.3.75; Kinesoft, Saskatchewan, Canada), and were included in the primary analyses if participants provided at least three days of valid data (including at least one weekend day). A valid day was defined as at least 500 min of data after excluding intervals of ≥60 min of zero counts, allowing up to two minutes of interruptions. Minutes spent in MVPA and mean sedentary time per day (SED) were derived from the accelerometry data using population-specific cut points for children [38] and adults [39].

### Demographic information

Parents provided demographic information via a questionnaire, including parent and child gender, date of birth, ethnic origin, and parental employment. As an indicator of socio-economic status, Indices of Multiple Deprivation (IMD) scores, based upon the English Indices of Deprivation [40], were assigned to each child based on their reported home postcode, where higher scores indicate greater levels of deprivation.

### Screen-viewing data

Parents were asked to complete questions about their child’s SV time on weekdays and weekend days. Separate questions were asked for the following SV devices: televisions; computers/laptops; and games consoles. For each device, parents were asked to report the time their child spent using it for (a) a normal weekday, and (b) a normal weekend day, with response options: none; 1 to 59 min; 1–2 h; 2–3 h; 3–4 h; 4 or more hours. Previous research indicates that assessing television viewing via parental response to a single question is moderately correlated (*r* = 0.60) with 10-days of television diaries among young children [41]. For analysis, a value was allocated to each of the response options as follows: none = 0 min; 1–59 min = 30 min; 1–2 h = 90 min; 2–3 h = 150 min; 3–4 h = 210 min; and 4 or more hours = 270 min.

### Data analysis

Interviews were transcribed verbatim and anonymised before being entered into QSR NVivo 10 (QSR International, Warrington UK) to facilitate analysis. Using the framework method, thematic content analysis was performed by two researchers, enabling themes to develop both inductively from the accounts (experiences and views) of participants and deductively from existing literature [42, 43]. Analysis involved several phases: familiarisation, coding, framework development, framework application, and interpretation. During familiarisation, transcripts were thoroughly read and re-read independently by two researchers to immerse themselves in the data. After discussion, an initial coding matrix was developed, reducing the data into codes and sub-codes, allowing constant comparison and refinement between researchers. The two researchers met regularly to ensure accuracy and consistency. Over-arching themes and sub-themes emerged, summaries produced, and for each theme representative quotes were extracted for reporting purposes. Final quotes were selected as being illustrative of several responses given by participants. For brevity, and to increase understanding about the child being discussed in each quote, information on interview number, parent gender, child gender, weekday television viewing (TV) time (perception of weekday SV level), weekend television time (perception of weekend SV level), and child mean MVPA minutes per day is provided after each quote.

Means and proportions were used to describe the characteristics of the interview sample. SV data was used descriptively, whereby the mean value for children’s time spent TV viewing, using a computer, or playing on games consoles on a weekday and weekend day, as reported by parents in the earlier questionnaire, was presented for each of the SV levels described by parents in the interview (e.g., low, medium, high).

## Results

The characteristics of the interview participants and their children are shown in Table 1. Thirty-one parents were mothers and 20 were fathers, with an average age of 41.2 years, a mean BMI of 25.8 kg/m^2^, and 94.1% were White British. The mean household IMD score was 11.5. Mothers are more likely to participate in research than fathers [44], so while a good proportion of interview participants were fathers (39.2%), it was unsurprising that a greater number of mothers participated. National data for England and Wales suggests that the average ages of mothers and fathers of nine year-old children born in 2007 are 38.3 and 41.3 years respectively [45], suggesting that mothers in the present study are slightly older than the national average. Mean parent BMI was slightly lower than the national average for adults in England (27.3 kg/m^2^) [46]. 91.8% of adults in South West England describe their ethnicity as White British [47], meaning our sample was slightly less diverse than the regional average. The four local authority areas (Bristol, Bath, South Gloucestershire, North Somerset) covered by the study have mean IMD scores ranging from 11.4 in South Gloucestershire to 27.2 in Bristol [40], suggesting the study participants were at the lower range of deprivation compared to their area averages. The average interview duration was 34.4 min (SD: 8.0 min, range: 18 to 55 min).

**Table 1:** Characteristics of the interview sample of parents (*N* = 51) and their children

	Parents		Children	
	Mean (SD)	%	Mean (SD)	%
Gender (% female)		60.8		51.0
Age (years)	41.2 (4.5)	–	9.0 (0.4)	–
Body mass index (BMI; kg/m^2^)^a^	25.8 (6.1)	–	0.01 (0.95)	–
Index of multiple deprivation	11.5 (9.7)	–	–	–
Moderate-to-vigorous physical activity (mins/day)	48.1 (21.5)	–	58.3 (17.4)	–
Sedentary time (mins/day)	568.3 (149.3)	–	451.9 (103.6)	–
Weekday SV (mins/day)				
Television watching	–	–	54.0 (41.6)	–
Computer use	–	–	34.8 (36.5)	–
Games console use	–	–	28.2 (50.5)	–
Weekend SV (mins/day)				
Television watching	–	–	123.6 (64.3)	–
Computer use	–	–	49.8 (57.1)	–
Games console use	–	–	58.8 (87.0)	–
Ethnicity				
White British	–	94.1	–	–
Other	–	5.9	–	–
Employment				
Full-time	–	45.1	–	–
Part-time	–	39.2	–	–
Unemployed/full-time parent	–	15.7	–	–

^a^Body mass index value for children is a BMI z-score, standardised for age and gender, based on the British 1990 Growth Reference [26]

### Importance of achieving a digital balance

The majority of parents believe it is important for their family to achieve a digital balance between SV and other activities (including physical activity). With SV on one side of the balance, parents identified spending quality family time together, interaction, physical activity, academic activities, and spending time outdoors as key factors in achieving this balance. In contrast, a few parents believed that SV was not an issue for their child, primarily because they did not feel their child engaged in SV for excessive periods, and/or because their child was physically active.

*‘I think, yeah, we do, we do encourage him to, you know, have a good balance of like screen viewing and academic and the physical activity’* ***[Int 48, male parent, boy, weekday TV 1-59 m (perception: low-medium), weekend TV 1-2 h (perception: medium), MVPA 49.3 min].***

*‘Again it's having that balance isn't it I think of having, allowing them to enjoy having screen time because it is good fun but also having the balance of doing other things as well, and going out together as a family, going for walks or bike rides or whatever it might be so it's just having that balance really.’* ***[Int 41, male parent, boy, weekday TV 1-59 m (perception: medium), weekend TV 2-3 h (perception: medium-high), MVPA 38.5 min].***

*‘So we don't like to have phones or tablets or anything at the table when we’re eating, so that, you know, for that 20 min or 40 min or however long it is, we’re actually looking at each other and talking to each other, rather than, you know, being otherwise engaged in something else.’* ***[Int 4, female parent, girl, weekday TV 1-2 h (perception: medium), weekend TV 2-3 h (perception: medium), MVPA 72.4 min].***

*‘I haven’t really had a problem of saying ‘oh turn that off we’re not watching that we’re doing something else’. And I mean yes if it became an issue then I probably, I wouldn’t, like I say I wouldn’t want them to sit in front of a screen for long periods but it’s not a problem I’ve encountered yet.’* ***[Int 2, female parent, boy, weekday TV 1-59 m (perception: low), weekend TV 1-2 h (perception: low-medium), MVPA 75.7 min].***

*‘I don’t think we’ve ever found it [managing SV] difficult because he’s always been keen to do physical things, like football, to go to the park to play with friends or what have you, kick around in the park or do one of his clubs or something. I think we appreciate we are lucky in that we never have to manage that in a very active way.’* ***[Int 44, male parent, boy, weekday TV 1-59 m (perception: medium), weekend TV 1-2 h (perception: medium), MVPA 100.6 min].***

### Sources of internal conflict regarding child SV

Parents in our sample expressed internal conflict regarding their child’s SV, whereby parents were uncomfortable with their children doing too much SV, but the usual response to restrict SV time is in conflict with contemporary SV norms and not wanting their children to be left behind. This internal conflict needs to be navigated if families are to achieve a digital balance. Four themes emerged from the interviews and analysis: 1) SV as the norm; 2) Positive aspects of SV; 3) Negative aspects of SV; and 4) Not all screens are equal. Each of the four themes is presented in detail below.

#### SV as the norm

Parents perceived that all children engage in regular SV, and that it is a normal part of the digital world their children are growing up in, where for some families the TV is ‘generally always on’ and used as a way for children to relax and ‘wind down’. Four parents reported that their family regularly engages with SV in the same room but on different devices. Numerous parents reported that due to SV being perceived as the norm, it was more difficult to limit the time their child spent SV.

*‘Me and my husband have discussed this, where he seems to think that our kids do it [screen viewing] more than they should, but I had to explain to him that it is the norm.’* ***[Int 39, female parent, boy, weekday TV 1-59 m (perception: medium), weekend TV 1-2 h (perception: high), MVPA 50.1 min].***

*‘I mean in the evening the TV seems to be generally always on. Um, and it does seem to be the default kind of relaxation tool for, well probably, probably everyone in the family I guess to a certain extent.’* ***[Int 19, male parent, girl, weekday TV 2-3 h (perception: medium), weekend TV 3-4 h (perception: high), MVPA 45.4 min].***

*‘And he watches Minecraft like all children, all the Minecraft channels...I think it's too much. But that's way it is these days, I guess.’* ***[Int 48, male parent, boy, weekday TV 1-59 m (perception: low-medium), weekend TV 1-2 h (perception: medium), MVPA 49.3 min].***

*‘But it's sometimes you'll find that we're all in the room together, but we might be watching something, but one of them will be watching something different on an iPad.’* ***[Int 42, male parent, boy, weekday TV 2-3 h (perception: medium), weekend TV 3-4 h (perception: medium-high), MVPA 61.9 min].***

#### Positive aspects of SV

Several parents believed that SV can be a useful tool for improving their children’s knowledge and technical skills, and thus improve their employability in later life. A few parents specifically mentioned that they did not want to restrict their child’s SV in fear that their child would be left behind in terms of their technological know-how. Parents reported other positives regarding child SV, for instance that SV can be used as a ‘virtual babysitter’, or as a tool to reward or punish child behaviour. A few parents also acknowledged that SV can be a sociable activity for children (e.g., playing video games online with friends).

*‘I think it’s more to do with not wanting the children to grow up ill-equipped to cope with the modern technological world...So they’ve got to be allowed to interact with these things, otherwise we’re giving them a bit of a disadvantage…they need to be technically minded so that they can sort of fill a huge gap the employment landscape.’* ***[Int 5, female parent, boy, weekday TV 1-59 m (perception: medium), weekend TV 1-59 m (perception: medium), MVPA 72.5 min].***

*‘I think we all – at times, every parent will plonk their child in front of the television so they can get on and do stuff.’* ***[Int 8, female parent, boy, weekday TV not reported (perception: medium), weekend TV not reported (perception: medium), MVPA 41.8 min].***

*‘But it’s incredibly useful on long car journeys, electronic devices, you know what I mean.’* ***[Int 10, female parent, girl, weekday TV 1-59 m (perception: medium), weekend TV 2-3 h (perception: high), MVPA 16.3 min].***

*‘We do sometimes use it as rewards as well, for doing good behaviour he might get some time on the Xbox.’* ***[Int 42, male parent, boy, weekday TV 2-3 h (perception: medium), weekend TV 3-4 h (perception: medium-high), MVPA 61.9 min].***

*‘Well, he gets a ban as a punishment, because I don’t want to ground him, because that does the opposite to what I want to achieve, so coming off the digital stuff is what I use mostly as a punishment.’* ***[Int 23, female parent, boy, weekday TV 1-59 m (perception: medium), weekend TV 2-3 h (perception: medium), MVPA 63.2 min].***

#### Negative aspects of SV

A number of parents mentioned how they think SV can have a negative impact on their children’s lives, suggesting multiple reasons including how they believe SV affects children’s behaviour, eyesight, posture, sleep, and reduces their opportunities for social interaction. Parents also believe that SV can be addictive, promote laziness, and can contribute to headaches, poor quality of life and obesity in children. One parent even referred to SV as causing their child to become ‘zombified’. A few parents referred to how ‘on-demand’ streaming facilitates binge-watching television shows, which can be a source of conflict for parents to negotiate. In contrast, a few parents did not mention any negative impacts of SV.

*‘Because I do think that there’s a real risk of a loss of children’s abilities to develop social interaction if they’re not having those opportunities to get away from screen time and just interact with their family’* ***[Int 4, female parent, girl, weekday TV 1-2 h (perception: medium), weekend TV 2-3 h (perception: low), MVPA 72.4 min].***

*‘They do go on Netflix to watch the odd series. And that’s when it gets into a bit of an unhealthy cycle because then they’ll binge on one show.’* ***[Int 5, female parent, boy, weekday TV 1-59 m (perception: medium), weekend TV 1-59 m (perception: medium), MVPA 72.5 min].***

*‘Well it’s [SV] making them more lazy and overweight isn’t it’.* ***[Int 23, female parent, girl, weekday TV 1-59 m (perception: medium), weekend TV 2-3 h (perception: medium), MVPA 63.2 min].***

*‘I’ve had it a couple of times with my daughter, she’s text me from upstairs and I’m downstairs, so I’m like, ‘No, if you want something you come and get it’.’* ***[Int 6, male parent, boy, weekday TV 1-59 m (perception: medium-high), weekend TV 3-4 h (perception: high), MVPA 58.1 min].***

*‘But sometimes it’s getting them out and getting them away from the screen in the first place. I think it’s hypnotic… We realised he was getting quite a lot of headaches and we thought, ‘Ooh actually no, I think we just let him do it too much’, which is when we started restricting it.’* ***[Int 36, female parent, boy, weekday TV 2-3 h (perception: high), weekend TV 3-4 h (perception: low), MVPA 51.4 min].***

*‘It can be addictive, and I think if children spend too long on screens I think it can have bad sort of health effects that can affect them badly. And causing, I think it makes them unhappy and depressed potentially.’* ***[Int 33, female parent, girl, weekday TV 1-59 m (perception: low), weekend TV 1-2 h (perception: low-medium), MVPA 48.5 min].***

*I: ‘Which is your priority, increasing physical activity or managing screen viewing?’*

*IV: ‘A bit of both really, so that he doesn’t strain his eyes and get a headache and, yeah, I am concerned about what he’s watching if I’m not supervising it.’* ***[Int 11, female parent, boy, weekday TV 1-2 h (perception: medium), weekend TV 2-3 h (perception: medium), MVPA 56.2 min].***

*‘There are so many violent games around these days, there are so many violent films around these days that you’ve just got to be a bit mindful, because obviously you don’t want them thinking that’s normal behaviour.’* ***[Int 6, male parent, boy, weekday TV 1-59 m (perception: medium-high), weekend TV 3-4 h (perception: medium-high), MVPA 58.1 min].***

#### Not all screens are equal

Several parents felt that not all SV was equal. Numerous parents perceived certain SV devices (e.g., tablets and games consoles) to have a particularly negative impact on children, due to the isolated and addictive nature of the activities, and how hard it can be to disengage children from these forms of SV. Time spent watching television was more acceptable, because parents viewed it as a more social activity where families can interact and discuss what they have watched. Many parents referred to regularly spending quality family time together watching films.

*‘TV we treat as obviously different, but we manage and monitor how long they're on electronic gadgets. So white screen [tablets] we call them... we just give them a sort of ten-minute warning, five-minute warning and then they're off…and they sit down and like watch the ‘Bake Off’ while I’m wiping up.’* ***[Int 51, male parent, girl, weekday TV 1-2 h (perception: medium), weekend TV 2-3 h (perception: medium), MVPA 70.5 min].***

*‘When my kids would be, they'd be… looking at separate tablet screens and then when they do watch something on TV it seems to me I'm just like wow that screen's brilliant, that's great, they're watching TV, because it's much more of a social thing. They'll be talking about what's happening on the screen, they'll be sitting together and yeah it just seems much more, much more pleasant activity.’* ***[Int 43, male parent, boy, weekday TV 1-59 m (perception: low), weekend TV 1-59 m (perception: low), MVPA 35.6 min].***

*‘On Friday we might watch a movie and we’ll try, if we’ve got enough time, to sit down with them and just watch something. So we’re resisting the temptation to get up and do stuff, but yes, it’s quite nice to sit down with them and watch altogether.’* ***[Int 38, male parent, boy, weekday TV 1-59 m (perception: medium), weekend TV 2-3 h (perception: medium), MVPA 90.8 min].***

*‘The television I don’t mind too much, but the computer thing, I think it can become so addictive, I don’t like the idea… There’s definitely a sort of mindset there that ‘I have to be on it and if I’m not, my world is going to fall apart’.’* ***[Int 9, female parent, girl, weekday TV 1-59 m (perception: low), weekend TV 2-3 h (no perception given), MVPA 59.5 min].***

Most parents preferred their child to engage in educational SV compared to other forms of SV, as they believe it to be more beneficial, with one parent even stating that they did not consider computer-based homework to be a form of SV. While it was reported that children mainly prefer other forms of SV, numerous parents stated that their child also enjoyed watching documentaries or researching topics online. Many parents actively encouraged educational SV, reporting that they have purchased equipment to facilitate educational SV. However, in one case this resulted in the child primarily using the computer for non-educational SV.

*‘Even though doing homework on a laptop is screen time…I’m not sure that I would really categorise it as that because it’s primarily homework, secondary a screen time thing whereas playing a game on an iPad is pure screen time in my opinion.’* ***[Int 50, male parent, girl, weekday TV None (perception: medium), weekend TV 1-2 h (perception: medium), MVPA 55.7 min].***

*‘We try to encourage them to watch more educational stuff. Depends what you class as educational. I mean, things like Blue Peter we just saw as part of kids’ TV when I was growing up, but now you look at it from an adult perspective it’s very educational; probably the most educational thing they’ll see.’* ***[Int 38, male parent, boy, weekday TV 1-59 m (perception: medium), weekend TV 2-3 h (perception: medium), MVPA 90.8 min].***

*‘You’re there thinking, ‘You need it for homework, so yeah we’ll get them a computer for homework.’ He doesn’t use it for his homework, does he? He uses it to play games. That’s completely defeated the object of why we got it.’* ***[Int 24, female parent, girl, weekday TV 1-59 m (perception: medium-high), weekend TV 2-3 h (perception: medium), MVPA 42.9 min].***

#### Parents’ descriptions of child SV level

Mean child weekday television viewing time, computer use and games console use (questionnaire data) by how parents described their child’s level of weekday SV (e.g., low, medium, high; interview data) are presented in Table 2 (weekend SV description categories are shown in additional file 2 Table S1). Parents described their child’s SV level as being low (25% and 14%), medium (50% and 34.9%) or high (10.4% and 20.9%) on weekdays and weekend days respectively. Mean reported weekday television viewing time and computer use were similar across description categories, while mean reported time spent playing on a games console varied from 10 min daily for children whose weekday SV was described by parents as ‘low to medium’, to 114 min daily for children who engaged in a ‘high’ level of SV. This suggests that children’s games console use may be an important influence for how parents describe their child’s level of SV. This finding was less pronounced for children’s weekend games console use. Compared to five mothers, no fathers described their child’s weekday SV level as being ‘high’. Similarly, for weekend SV, seven mothers and only two fathers described their child’s level of SV as ‘high’, suggesting that fathers may be less likely to describe to their child’s SV as ‘high’ than mothers. Most parents based their perceptions of their child’s SV level on comparisons with the SV behaviours of children’s friends, while some parents quantified how much SV their child does, in terms of programmes watched or hours spent engaged with SV.

**Table 2:** Parents’ descriptions of their child’s weekday SV time compared to numerate levels reported in questionnaire

Description^a^	No. of parents	Parent gender	Child gender	Daily minutes TV viewing Mean (SD)	Daily minutes computer use Mean (SD)	Daily minutes games console use Mean (SD)
Low	12	8 mothers 4 fathers	6 girls 6 boys	30.0 (22.2)	22.5 (13.6)	12.5 (15.4)
Low-medium	3	2 mothers 2 fathers	3 girls 1 boy	30.0 (0.0)	10.0 (17.3)	10.0 (17.3)
Medium	24	11 mothers 12 fathers	12 girls 11 boys	70.0 (45.7)	46.3 (37.5)	16.3 (26.5)
Medium-high	4	2 mothers 2 fathers	2 girls 2 boys	30.0 (0.0)	37.5 (75.0)	60.0 (73.5)
High	5	5 mothers 0 fathers	2 girls 3 boys	66.0 (53.7)	30.0 (36.7)	114.0 (100.4)
Could not categorise	3	3 mothers	1 girl 2 boys	60.0 (42.4)	15.0 (21.2)	15.0 (21.2)

^a^Parents responded to the interview question: ‘If you were to describe your child’s level of screen viewing as on weekdays as low, medium or high, which one would you pick?’

*I: ‘So if you were to describe her level of screen-viewing during the week as low, medium or high, which one would you pick?’*

*IV: ‘High, definitely high, yeah, yeah… because, I would say she’d on her phone or iPad, I would say probably every morning before she goes to school, and then certainly on an evening I would say it’s from about seven o’clock till eight, till she goes to bed, maybe till half eight.’* ***[Int 25, female parent, girl, weekday TV 1-59 m (perception: high), weekend TV 1-2 h (perception: high), MVPA 83.9 min].***

*‘It’s [weekday SV] low, it’s definitely low, but then that’s enforced by me. It’d be higher if I let her, but it’s definitely low.’* ***[Int 28, female parent, girl, weekday TV none (perception: low), weekend TV 1-2 h (perception: low-medium), MVPA 69.9 min].***

Some parents found it difficult to categorise their child’s SV level, suggesting they were unaware of how much SV was the ‘norm’. Parents reported being unsure of the accuracy of their perceptions, uncertain whether their interpretation of SV level matched others, and one parent even stated how their child’s friends described their SV levels very differently to descriptions by their parents.

*‘I would probably say it was medium. It’s difficult. I don’t really know how much other children actually screen view really to say whether it’s good, bad or indifferent. It’s hard to compare because I don’t know whether two hours is excessive or is actually quite a lot less’.*

***[Int 4, female parent, girl, weekday TV 1-2 h (perception: medium), weekend TV 2-3 h (perception: low), MVPA 72.4 min].***

*‘I think she could watch a lot less which obviously would be low because she does watch like a programme a day...I don’t know if you would call that low or whether I would call low...I’d say she’s between low to medium’* ***[Int 35, female parent, girl, weekday TV 1-59 m (perception: low-medium), weekend TV 1-59 m (perception: low), MVPA 71.5 min].***

*‘I’ve no idea [laughs]. I mean, I can give an answer but I wouldn’t be accurate. I don’t know. I would say it’s probably low to medium’* ***[Int 46, male parent, girl, weekday TV 1-2 h (perception: low-medium), weekend TV 1-2 h (perception: medium), MVPA 58.5 min].***

*‘I mean it’s hard to know what other families do, because the mums will say, ‘Oh they don’t play on it’, but then the children come round and they say, ‘Oh, I’m allowed to go on Xbox until this much time’.’* ***[Int 23, female parent, boy, weekday TV 1-59 m (perception: medium), weekend TV 2-3 h (perception: medium), MVPA 63.2 min].***

## Discussion

The data in this paper demonstrate that many parents feel internally conflicted about their child’s SV, on one hand they recognise the benefits of SV and do not want their child to be left behind, in terms of their technological know-how. At the same time, they appreciate some of the negative aspects of SV (e.g., effects on eyesight, behaviour, isolation, obesity), and thus feel it is important to achieve a digital balance, in order to spend more quality family time together and improve child health. Our data showed that parents believe that SV can be a useful educational tool, improve employability, be a way for children to relax, and act as an ‘electronic babysitter’, similar to previous research with Canadian parents of pre-school children [22] and 5–17 year-olds [23]. Research has also indicated that children whose parents allow SV to keep them quiet, and provide or restrict SV time to reward or punish behaviour are more likely to engage in SV for over two hours per day [48]. Therefore, strategies for navigating this internal conflict are needed, for example helping parents to understand the negative effects of SV, downplay the importance of SV for children, and suggesting alternative ways to spend quality family time together.

Many parents place different values on forms of SV, and not all SV is considered equal. One parent even stated that they do not class computer-based homework as SV. In their updated recommendations, the AAP have recognised this and make a distinction between educational and entertainment SV, encouraging parents to limit the entertainment side of media use while being more lenient on educational SV [12]. Interestingly, some parents reported being happy to allow their child watch television, but have stricter guidelines about tablet use, primarily due to the isolated nature of tablet activities causing children to become ‘zombified’. It may be difficult for parents to communicate a long list of acceptable and less acceptable SV types, times, and sources to children, because such messaging would be complex and difficult to maintain consistency, therefore, parents tend to create simple blanket rules, which are harder to enforce because the target of the rule is more complex. This highlights a need to educate parents about the risks associated with all types of SV, and encourage them to find ways to limit forms of entertainment SV, regardless of device.

Child SV time, as reported by parents in the earlier questionnaire, varied considerably by how parents described their child’s SV level (e.g., low, medium, high) during the interview. These differences were more pronounced on weekends, and fewer fathers viewed their child’s SV level as ‘high’ compared to mothers. One father perceived his son’s weekend level of SV to be ‘medium’, despite reporting that their child engages in SV for over eight hours per day, far exceeding the previous AAP child SV recommendations of two hours per day [13]. Most parents based their descriptions of child SV level on knowledge of their child’s friends’ SV levels, with fewer basing it on the time their child spends SV. Therefore, parents may consider their child’s level of SV to be normal because ‘that’s the way it is these days’. Parents have their children’s best interests at heart and may believe achieving a digital balance is important, however, this does not necessarily mean they recognise when their child’s SV level is problematic. While it is important to keep SV recommendations flexible to adapt to the rapidly-changing technological environment, strategies need to be implemented to help parents understand how much SV is excessive and how to achieve an appropriate balance of different activities.

### Strengths and limitations

A main strength of the study is the recruitment of a relatively large sample of parents, with a good level of variation in socio-economic position, including 20 fathers, a group that are known to be difficult to engage in research [44]. As this qualitative research was embedded within a larger cohort study, we were able to utilise parent-reported measures of child SV behaviour from the main quantitative dataset, resulting in a rich and unique dataset that has provided novel insights into how parents perceive their child’s level of SV. Moreover, the robustness of the data collection and analysis process has provided a rigorous evaluation of the area, and there was clear saturation of information in the analyses. The study is, however, limited by the self-reported nature of the SV measures, because there are no objective measures of SV available for use in large cohort studies. The ordinal nature of the SV behaviour questionnaires enabled parents to report behaviours easily, however, it also meant that it was not possible to calculate an exact total SV score. Additionally, while some parents may have considered night-time SV behaviours within their estimation of total SV time, night-time SV was not explicitly measured within the present study, therefore, total SV time might be underestimated. Night-time SV has previously been associated with health outcomes (sleep duration, body weight, diet quality and physical activity) in a Canadian study of 3398 children of a similar age [49]. Future research is needed to develop validated measures of child SV (both objective and reported measures). This study is also drawn from one large city in the Southwest of England and the surrounding area, and while the participants were mostly similar to regional and national averages, our ability to extend findings to other settings and countries is limited.

## Conclusions

Parents feel conflicted about the positive and negative aspects of SV, having concerns about how SV can affect their child’s health, while simultaneously not wanting their child to be ill-equipped to cope with the modern technological world. Despite this, most parents feel it is important for their family to achieve a digital balance. Additionally, parents do not consider all forms of child SV to be equal, with television watching and homework-based SV perceived as more acceptable than tablet use. Therefore, researchers and policy makers need to develop innovative ways to support families to achieve a digital balance, considering the reasons why SV is viewed positively by many parents, how parents view some forms of SV as more acceptable than others, and the rapidly changing technological environment.

## Additional files

Additional file 1: Interview Guide. The interview guide used to conduct the qualitative interviews. (DOCX 29 kb)

Additional file 2: **Table S1.** Parents’ descriptions of their child’s weekend SV time compared to numerate levels reported in questionnaire. A table displaying parents’ descriptions of their child’s weekend screen viewing time compared to numerate levels reported in the questionnaire. (DOCX 13 kb)

## Abbreviations

AAP: American Academy of Pediatrics
BMI: Body mass index
IMD: Index of multiple deprivation
Int: Interview
MVPA: Moderate-to-vigorous-intensity physical activity
SD: Standard deviation
SED: Sedentary
SV: Screen-viewing
TV: Television
