# Supplementary material for: Examining the challenges posed to parents by the contemporary screen environments of children: a qualitative investigation
Source: BMC Pediatr. 2018 Apr 7;18:129. doi: 10.1186/s12887-018-1106-y (PMC5889845; doi:10.1186/s12887-018-1106-y)
Supplement: Supplementary file 1 — Interview Guide. The interview guide used to conduct the qualitative interviews. (DOCX 29 kb) [file 12887_2018_1106_MOESM1_ESM.docx]

**Year 4 – B-Proact1v Qualitative Interview Guide**

Welcome them.

Thank you for agreeing to take part in this interview, your views and opinions are really important to us. In the interview today, we are going to be talking about your perceptions of your child’s physical activity and screen-viewing behaviours, try to understand more about your experiences of physical activity and screen-viewing during your own childhood, and how this compares to your child’s experiences, how families strike a ‘digital balance’, how we might best change or manage physical activity or screen-viewing behaviours, as well as understanding what has changed in the last three years and what your expectation for your child are going forward.

We are really interested in your honest opinions, we are not here to judge you, and we do not want you to feel like you should answer any of the questions in a certain way, there are no right or wrong answers, and as much detail you can give on the topics as possible is really appreciated.

- Before we get started, I’d like to tell you that I will be **recording the conversation** to help us remember what you said.
- You can ask for the recording to be stopped at any time.
- After we have written a report about all the opinions we have heard from the parents taking part, the recordings will be destroyed
- We will also change any names or identifying information so none of the information that is written down and recorded can be connected to you in any way
- Please remember that you can interrupt the interview at any point if you need to.
- If you do not want to answer a question please say so.
- Are you ok to go ahead now?

If the parent says yes the recording will start. As noted on the information sheet the interview will be recorded.

- For the recording, can you please confirm that you have been given information explaining about the study and that you understand what this project is about?
- Can you please confirm that you have had the opportunity to ask questions and discuss this study?
- Can you please confirm that you are aware that the interview data will be stored anonymously and securely for 20 years?
- Can you please confirm that you are aware that you are free to withdraw your data from the study at any time up to three weeks after this interview has taken place **[X DATE]**, and that you do not need to give a reason for withdrawing?
- And finally, can you please confirm that you are happy to take part in the interview?

| **Ice-breaker:**  **Can you tell us the name of your child in year 4 and what their favourite physical activity/physically active thing to do is?**  **We want to focus on your year 4 child, but could you tell us who else lives in the household, does (child name) have any brothers/ sisters (and how old are they?)** | | | | |
| --- | --- | --- | --- | --- |
| **Part one: Some children are more or less physically active than others and some children spend more or less time in front of a screen than others, whether that is a TV, computer, tablet (e.g., iPad), mobile phone, games console or hand-held device.** | | | | |
| **Check** | **Questions** | **Explanation** | **Prompts** | **Theme** |
|  | **If you were to describe your child’s level of physical activity as low, medium or high, which one would you pick?**  **Can you tell me more about why you picked ‘X’?**  **Compared to other children their age, how active do you feel your child is?** | (PA is anything that gets the body moving –it can be in the form of structured exercise or it can be free play, running around the garden, walking to school)  E.g., their friends at school | Why? (do you think that)  Why? |  |
|  | **What about screen time? If you were to describe your child’s level of screen viewing on weekdays as low, medium or high, which one would you pick?**  **- Can you tell me more about why you picked ‘X’?**  **What about weekend days? Would you describe their screen viewing on weekend days as low, medium or high?**  **- Can you tell me more about why you picked ‘X?**  **Compared to other children their age, how much screen viewing does your child do per day?** | We use the phrase ‘screen viewing’ as a catch-all phrase to include: computers, laptops, games consoles, ipads, mobile phones, not just TV  Why? How do you know this? | What is the reason you feel this way? Do you feel SV is a good or bad thing? |  |
|  | *****If not already covered***(How do you feel about the amount of time your child spends screen viewing?)** | Why? |  |  |
|  | **Do you have times where screen-viewing is OK (e.g., to chill out/relax, to watch an educational programme, or just to entertain them) and other times when you’d prefer your child to do something else?**  **Do you mind what content they watch?**  **Do you regularly engage in educational-based SV with your child?**  **Do you regularly engage in non-educational SV with your child?** | If so, why? Why not?  E.g., homework, educational TV.  E.g., TV for downtime, family bonding, TV on in background | How has this changed since your child was in Year 1?  If so, why & how often?  If so, why & how often? |  |
|  | **Is it important to your family to find / achieve a “digital balance”?**  **(By digital balance, we are talking about your child having a balanced lifestyle that includes SV but also includes a good balance of other activities and time away from SV)**  **Have you (or your partner) ever encouraged your family to take a break from screen viewing? (e.g., or a “digital detox”?)** | If so, why?  If so, how often? For how long (e.g. one day, weekend, weeks, etc)? Was it successful? How did it go, what did you do instead, how did you children react? | Health reasons? Safety concerns? Family time, perceptions of “quality time”  What are the “other activities” in the balance that you want your child to do |  |
|  | **If you were to encourage your child to take a break from screen viewing, what do you encourage them to do instead?**  **Is physical activity a realistic substitute for screen viewing for your child?** | E.g., PA, reading, creative activities, music  If so, why? Or why not? | Is it helpful/unhelpful? |  |

| **Part two: This section aims to explore what you would want and need in order to make changes to the physical activity and screen-viewing behaviour of your family** | | | | |
| --- | --- | --- | --- | --- |
| **Check** | **Questions** | | **Prompts** | **Theme** |
|  | **Is increasing physical activity among your family important to you?**  **Is managing screen viewing among your family important to you?**  **If you had to pick one of these, which is your priority?** | Could be together as a family (e.g., cycle ride) or separately (e.g., take child to swimming).  How achievable/ possible is it?  E.g, increase PA or manage SV? | How achievable/ possible is it? Why? In what way?  Why? In what way?  Why? |  |
|  | **You’ve mentioned that you would like to change X [either PA or SV behaviour], (do you think that you could make that change now?)**  **If not, what information or resources do you need to help you make that change?**  **Where would you go to get this information or plan? How would you approach the change?** | If so, how?  E.g., advice, knowledge, help with conflict resolution, setting limits, engaging other parent, influence of siblings.  E.g., Internet? Friends? Child’s school? Make it up? | Why? How do you know this?  Why? |  |
|  | **When we interviewed parents previously, they suggested some approaches to increase children’s physical activity (please see 1)**  **Are there any approaches that you think would work/not work?**  **They also suggested ways that parents could manage their child’s screen viewing (please see 2). Are there any approaches that you think would work/not work?** | Why? Do you think they would work with your child? Why/why not?  Why? Do you think they would work with your child? Why/why not? | Can you think of any other approaches that you believe would work with your child?  Can you think of any other approaches that you believe would work with your child? |  |

| **Part three: I now want you to think about your own childhood, and how the physical activity and screen viewing environment might have been different to what your child experiences now.** | | | | |
| --- | --- | --- | --- | --- |
| **Check** | **Questions** | | **Prompts** | **Theme** |
|  | **Compared to experiences from your own childhood, how do you feel that physical activity is different for your Year 4 child now?** | Do children now do more or less PA? Preferences for PA? Types of PA different? Is PA more/less structured or more/less free-play? | Why? What impact do you think this has had on your child? |  |
|  | **When you were a child, do you feel that physical activity was seen as important or valuable? If so, in what ways? If not, why not?**  **Do you think that this level of value (or importance/lack of importance) is different for your child? Why or why not?** | How did this make you feel about being active?  Did you think about it as PA or were you just playing out and having fun?  More or less important/valuable?  ** Probe into this** | If appropriate – probe about imagination and play – e.g., making up games (dens, bike circuits)  Does your child use their imagination during play or is it more structured at play centres etc.,? |  |
|  | **Now thinking about screen viewing, compared to experiences from your own childhood, do you think the screen viewing environment is different for your child now? If so, how?** | Do children now do more or less SV? Importance placed on SV? Preferences for SV? Types of SV different? Necessity of SV changed? | Why? What impact do you think this has had on your child? |  |
|  | **[If they said that the screen-viewing environment has changed since their childhood for question above] The screen viewing environment has changed since you were a child, how do you feel about the environment that your child is growing up with?** | Do you feel under pressure to keep up with changes in technology, and allowing your child access to screen viewing devices? | If so, who by? (E.g., child, school, family members)  Does it cause any conflict/tension in the household? Why? |  |

| **Part four: Finally, we are interested in how families change across time, in terms of how they manage physical activity and screen viewing behaviours. We interviewed a sample of parents three years ago, when your child was in Year 1 at school.** | | | | |
| --- | --- | --- | --- | --- |
| **Check** | **Questions** | | **Prompts** | **Theme** |
|  | **Looking back across the last three years, what has changed with your Year 4 child – in terms of their physical activity?**  **What about their screen viewing?** | More or less active? More or less interested? Involved in different activities?  More or less SV? Has their interest changed? Types of SV engaged in? Easier or more difficult to manage? | Why? |  |
|  | **We hope to interview a sample of parents again in two years, when the children are in Year 6.**  **What are your expectations for the next two years – in terms of your child’s physical activity?**  **What about their screen viewing?**  **Do you have any plans for how you might manage their screen viewing going forward?** | E.g., hopes and fears? New activities. Expect them to be more/less active?  E.g., hopes and fears? New SV behaviours? Expect more or less SV?  E.g., setting limits, suggesting alternatives. | Why? (do you think this)  Why? (do you think this)  Why? (have you chosen this) |  |

| **Part five: We’ve just completed a study looking at the benefits of delivering after-school clubs to increase physical activity and learn new skills. We’d like to get your views on after-school clubs and whether you think they would be useful to your child.** | | | |
| --- | --- | --- | --- |
| **Check** | **Questions** | **Prompts** | **Theme** |
|  | **Has your child attended after-school clubs in the past? What was it about the club that they enjoyed or did not enjoy?** |  |  |
|  | **If your child has attended after-school clubs in the past, what was it that made them want to take part?** |  |  |
|  | **How could we interest your child in taking part in an active after-school club?** |  |  |
|  | **What things would prevent your child from attending an active after-school club?** |  |  |
|  | **We want to run a fun, free and inclusive active after-school club in your child’s school. The sessions will focus on having fun and being active, they will not require any previous skills or experience, and your child will be able to try new activities and learn new skills. Based on this description, what things would you emphasise to your child in order to get them to attend?** |  |  |

**CLOSING** (2-3 minutes)

- Is there anything else you’d like to tell us about the things we talked about today?
- Do you have any questions for me?
- We appreciate you sharing your thoughts and opinions with us!

*Take down/check personal details for sending them the £10 Love2Shop voucher before ending the call – stop recording right at the end*

**Interviews: Follow-up responses which are neutral.**

**To end of a point before moving on to the next:**

‘That’s interesting, thank you’

‘Thank you for that information’

‘Ok, I understand, thank you’

‘That is useful to know’

‘Thank you for that. Let’s move on to the next section’

‘I see, that is very useful information. So moving on, can we talk about…..’

**To probe/prompt a point:**

‘Why do you think that is?’

‘Could you give me an example?’

‘What did you try?’

‘Can you expand on that?’

That’s interesting, can you tell me some more/explain that in more detail?’

‘Is that always the case?’
