# Supplementary material for: Examining the challenges posed to parents by the contemporary screen environments of children: a qualitative investigation
Source: BMC Pediatr. 2018 Apr 7;18:129. doi: 10.1186/s12887-018-1106-y (PMC5889845; doi:10.1186/s12887-018-1106-y)
Supplement: Supplementary file 2 — Table S1. Parents’ descriptions of their child’s weekend SV time compared to numerate levels reported in questionnaire. A table displaying parents’ descriptions of their child’s weekend screen viewing time compared to numerate levels reported in the questionnaire. (DOCX 13 kb) [file 12887_2018_1106_MOESM2_ESM.docx]

**Supplementary Table 1 Parents’ descriptions of their child’s weekend SV time compared to numerate levels reported in questionnaire**

| Description* | No. of parents | Parent gender | Child gender | Daily minutes TV viewing  Mean (SD) | Daily minutes computer use  Mean (SD) | Daily minutes games console use  Mean (SD) |
| --- | --- | --- | --- | --- | --- | --- |
| Low | 6 | 3 mothers  3 fathers | 3 girls  3 boys | 90.0 (93.0) | 15.0 (16.4) | 75.0 (111.8) |
| Low-medium | 6 | 5 mothers  1 fathers | 3 girls  3 boys | 120.0 (32.9) | 35.0 (58.2) | 50.0 (45.2) |
| Medium | 15 | 7 mothers 8 fathers | 8 girls  7 boys | 134.0 (73.4) | 60.0 (77.7) | 46.0 (80.1) |
| Medium-high | 7 | 2 mothers  5 fathers | 5 girls  2 boys | 98.6 (64.1) | 30.0 (30.0) | 25.7 (32.1) |
| High | 9 | 7 mothers  2 fathers | 4 girls  5 boys | 143.3 (46.9) | 60.0 (47.4) | 103.3 (125.6) |
| Could not categorise | 8 | 7 mothers  1 father | 3 girls  5 boys | 132.9 (57.1) | 77.1 (48.6) | 55.7 (87.3) |

* Parents responded to the interview question: ‘If you were to describe your child’s level of screen viewing as on weekend days as low, medium or high, which one would you pick?’
